# Controlled ovarian stimulation with r-FSH plus r-LH vs. HMG plus r-FSH in patients candidate for IVF/ICSI cycles: An RCT

**Published:** 2017-07

**Authors:** Ensieh Shahrokh Tehraninejad, Mina Farshbaf Taghinejad, Batool Hossein Rashidi, Fedyeh Haghollahi

**Affiliations:** *Vali-e-Asr Reproductive Health Research Center, Tehran University of Medical Sciences, Tehran, Iran.*

**Keywords:** Ovulation induction, Recombinant-FSH, Recombinant-LH, Human menopausal gonadotropin, IVF, ICSI

## Abstract

**Background:**

Different combination of gonadotropin preparation has been introduced with no definite superiority of one over others in in vitro fertilization (IVF), but individualized regimens for each patient are needed.

**Objective:**

The aim of the present study was to investigate the effect of controlled ovarian stimulation with recombinant- follicle stimulating hormone (r-FSH) plus recombinant-luteinizing hormone (rLH) versus human menopausal gonadotropin (HMG) plus r-FSH on fertility outcomes in IVF patients.

**Materials and Methods:**

This is a randomized clinical trial study that was performed from October 2014-April 2016 on 140 infertile patients with a set of inclusion criteria that referred to infertility clinics in Vali- asr and Gandhi Hospital in Tehran. The women were randomly divided into two treatment groups. The first group (n=70) received rFSH from the second day of cycle and was added HMG in 6^th^ day and the 2^nd^ group (n=70), received rFSH from the second day of cycle and was added recombinant-LH in 6^th^ day. Then ovum Pick-Up and embryo transfer were performed. In this study, we assessed the outcomes such as; chemical and clinical pregnancy rate, live birth and abortion rate.

**Results:**

Number of follicles in ovaries, total number of oocytes or M_2_ oocytes and quality of fetuses has no significant differences between two groups (p>0.05). Total number of fetuses were significantly higher in patients who received rFSH + HMG (p=0.02). Fertility outcomes consisted of: live birth rate, chemical pregnancy and clinical pregnancy rate were higher in rFSH + HMG group in comparison to rFSH +r-LH group (p<0.05).

**Conclusion:**

It seems that in IVF patients, HMG + rFSH used for controlled ovarian hyperstimulation have better effects on fertility outcomes, but in order to verify the results, it is recommended to implement studies on more patients.

## Introduction

To date, different gonadotropin preparations have been introduced for controlled ovarian stimulation (COS) in pituitary-suppressed patients undergoing in vitro fertilization/intra cytoplasmic sperm injection (IVF/ICSI) procedures (1). Considering the fact that every individual patient has specific infertility reasons, demographic and medical profiles necessitate the use of individualized regimens in each patient which should be based on the physiology of normal pregnancy (1). In this regard, many studies have been published about the effect of recombinant luteinizing hormone (rLH) in COS. It was shown that Luteinizing Hormone (LH) modulates folliculogenesis by reducing the number of small or intermediate size follicles (2, 3). 

However, results of studies on the sufficiency of endogenous LH levels or the need for adding LH activity in pituitary-suppressed patients are controversial. LH activity can be administered in different forms, either adding r-LH to recombinant follicle stimulating hormone (rFSH) or using highly puriﬁed human menopausal gonadotropine (hMG) which provides follicle stimulating hormone and exogenous LH activity (1, 2, 4, 5). Although Hill and Alvigi showed superiority of adding exogenous LH to rFSH over FSH alone in terms of increased number of mature oocytes, good quality zygotes and higher implantation rates but other investigators reported no improvement in the outcomes when exogenous LH is added (6-9).

The aim of the present study was to investigate the rFSH+rLH vs. HP-hMG+ rFSH on fertility outcomes (pregnancy rate, abortion and live birth rate) in IVF patients.

## Materials and methods

**Study design**

**Figure1:**
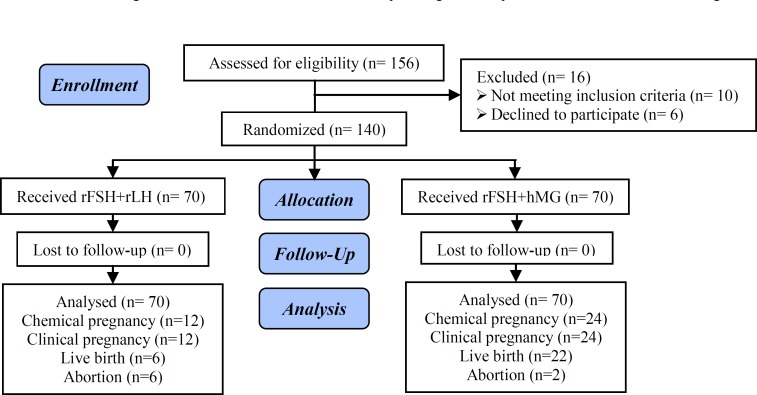
CONSORT flow diagram

This was a randomized controlled trial which was performed from October 2014-April 2016 at the Infertility Departments of Vali-e-Asr and Gandhi Hospital. Participants were allocated by the clinic secretary to one of two groups by simple random sampling, using a random numbers table. The clinician, ultra sonographer, embryologist and statistician were not blinded. From among 156 patients undergoing assisted reproductive technology cycles during the study period, 140 women were included with the following criteria; aged 20-35 yr, male factor, Tubal or unexplained infertility, regular menstruation cycle between 21 and 35 days, normal function of uterus according to hysterosalpingography, hysteroscopy or transvaginal ultrasonography, normal ovaries according to transvaginal ultrasonography during past 6 months prior to study and compatible with normal adnexa and normal ovarian anatomy, and serum FSH level less than 8 IU/l (Figure 1).

All women showed no recognizable endometriosis according to symptoms and clinical examination in transvaginal ultrasonography or diagnostic laparoscopy. All women had a history of unexplained infertility normal ovulatory function and normal semen analysis according to the World Health Organization criteria (10). Patients with other ovulation disorders such as hypo and hyper-gonadotropic, hypogonadism, hyper-prolactinemia, thyroid disorders, ovarian or adrenal neoplasms, Cushing syndrome, previous history of systemic diseases such as endocrine and metabolic disorders and a previous history of inappropriate ovarian response to stimulation with gonadotropins (poor responders), prior history of more than 3 unsuccessful IVF, and any malformation of sexual organs were excluded. Patients were divided into two groups. 

**Treatment protocol**

Baseline FSH, LH, anti Mullerian hormone (AMH) prolactin, thyroid stymulating hormone and testosterone serum levels were measured for all patients in their previous cycles. All patients received oral contraceptive from day 5 of menstruation cycle and underwent pituitary down regulation receiving a once daily subcutaneous dose of 0.1 mg (Decapeptyl, Ferring, USA) from day 21 in addition to a short-acting gonadotropin releasing hormone (GnRH) analog (Buserelin ®; Suprefact, Hoechst, AG-Germany) per day (administered subcutaneously) from the 21^st^ day of their cycles with oral contraceptive pills pretreatment. All the following gonadotropins were injected subcutaneously by patients.

After stopping oral contraceptive pills for pituitary suppression when the bleeding occurred, the patients were randomly allocated to group 1, (n=70) who received recombinant FSH (Gonal-F, Serono, Switzerland) (75 IU per ampoule) started on day 2 of menstruation and then after six days, hMG (Merional, 75 Iu, s.c) was added. Administration of HCG (Profasi®, Laboratoires Serono S.A.), 10,000 IU i.m. was done, based on ovarian response as assessed by sequential vaginal ultra sonography until the leading follicle had reached a diameter of 18 mm.

Group 2 (n=70) were treated with recombinant FSH (Gonal-F, Serono, Switzerland) (75 IU per ampoule) and after six days the recombinant LH (Levuris, Serono, Switzerland), 75 IU subcutaneously (s.c.) was added. Dose for HMG or rFSH were dependent on age and follicular response, between 150225 units. Vaginal sonoghraphic exam was performed and in case of appropriate response, the patients underwent sonography every other day until they had at least two follicles ≥18 mm and at least two other follicles with a diameter >17 mm. Ovulation was induced by administration of HCG (Profasi®, Laboratoires Serono S.A.), 10,000 IU i.m. Endometrial thickness were measured on HCG injection day.

Oocyte pickup was performed 34-36 hr following HCG administration. After the ICSI procedure, embryos were scored according to the morphologic appearance of their blastomers and fragmentation (11). Embryo transfer was performed on day three of ovum pickup and 2-3 embryos being transferred per patient by the sono-opaque catheter (Cook Medical, Ireland LTD) under sterile condition. In all patients, the luteal phase was supported by Cyclogest (Actover, Alpharma, England) with a vaginal progesterone at a dose of 400 mg/Bid, which started from the day of oocyte retrieval. In cases where chemical pregnancy was detected 2 wk following embryo transfer, clinical pregnancy was confirmed with ultrasound examination with the appearance of a gestational sac 6 wk thereafter. 

**Study outcomes**

Previous documents were used to extract data. Basic information such as age, weight and height, marriage years, duration of infertility, underlying causes of infertility, regularity or irregularity of menstruation cycle, serum levels for FSH, LH, Thyroid Stymulating Hormone, AMH and prolactin, and results for previous imaging studies such as hysterosalpyngography were recorded. Type of protocol was extracted from past records too. Number of ovum in right and left ovary, number of oocytes and oocytes of metaphase 2, number of fetuses and related type, birth or abortion also extracted from the records. We assessed the chemical and clinical pregnancy, live birth rate, abortion and ovarian hyper stimulation in this study.

**Ethical consideration**

After being accepted by the Research Committee of Tehran University of Medical Sciences and also obtaining ethical approval from the Faculty of Medicine Ethics Committee, written inform consent was obtain from participants.

**Statistical analysis**

Statistical analysis was performed using Statistical Package for the Social Sciences, version 17 (version 17.0, SPSS Inc, Chicago, Illinois, USA, SPSS). Qualitative variables assessed using Chi-squared test, normally distributed quantitative variables by student’s t-test and non-parametric analysis were done using Mann-Whitney U test. Normal distribution assessed using Shapiro-Wilk test. All the cut-off for statistical significance presumed 0.05.

## Results

70 patients in each group were selected. There were no significant differences between basic parameters in these 2 groups (Table I). The most common underlying cause of infertility was related to male factor. There were no significant differences between the underlying factors (Table II). The number of follicles in right and left ovary, total number of oocytes or M2 oocytes and quality of fetuses has no significant differences between groups; but, total number of fetuses was significantly higher in patients who received rFSH and HMG (Table III). 

Fertility outcomes in different treatment groups including live birth rate, chemical pregnancy and clinical pregnancy were all better in rFSH and HMG group in comparison to rFSH and LH group. Also, there was no difference in number of abortion between two groups (Table III). There was no ovarian hyper stimulation and ectopic pregnancy in two groups.

**Table I:** Basic demographic, clinical and obstetrics information of patients

		**rFSH + HMG (n=70)**	**rFSH + LH(n=70)**	**p-value**
Oocyte retrieval age, yr, mean (SD)		37.60 (7.44)	38.82 (8.44)	0.58
Duration of infertility, yr, mean (SD)		6.03 (4.17)	4.31 (3.03)	0.30
Type of infertility, n (%)				
	Primary	36 (54.5)	30 (45.5)	0. 56
	Secondary	20 (37.0)	34 (63.0)	
Serum level of AMH, ng/mL, mean (SD)		2.89 (3.96)	2.01 (1.93)	0.23
Serum level of LH, mIU/mL, mean (SD) *		6.35 (6.59)	4.63 (3.52)	0.08
Serum level of FSH, mIU/mL, mean (SD)		7.02 (3.00)	6.46 (3.63)	0.34
Serum level of TSH, U/mL, mean (SD)		2.33 (1.45)	2.09 (1.74)	0.40
Serum level of Prolactin, ng/mL, mean (SD) *		33.18 (74.61)	87.30 (214.60)	0.07

p-value refers to student T-test or Chi-squared test, when appropriate.

rFSH: recombinant follicle stimulating hormone

HMG: Human menopausal gonadotropin

LH: luteinizing hormone

AMH: Ani- mulerian hormone

FSH: Follicle-stimulating hormone

TSH: Thyroid- stimulating hormone

**Table II:** The underlying factors of infertility

	**rFSH + HMG (n=70)**	**rFSH + LH (n=70)**	**p-value**
Male factor	45.9%	51.1%	0.54
Female factor	42.9%	43.5%	
Unexplained	11.2%	5.4%	

Chi-squared test

rFSH: recombinant follicle stimulating hormone

HMG: Human menopausal gonadotropin

LH: luteinizing hormone

**Table III:** Treatment outcomes in patient in 2 groups

		**rFSH + HMG (n=70)**	**rFSH + LH (n=70)**	**p-value**
Total number of oocytes, mean (SD)		10.74 (6.34)	10.06 (5.15)	0.48
Total number of M2 oocytes, mean (SD)		9.36 (6.10)	8.03 (4.80)	0.15
Total number of embryo, mean (SD)		6.97 (4.65)	5.29 (4.46)	0.03
Total number of transferred embryo, mean (SD)		2.26 (0.69)	2.00 (0.87)	0.06
Quality of transferred embryo (n, %)		68	58	0.12
	A	44 (62.85)	34 (48.57)	
	A-B	14 (20.0)	20 (28.57)	
	B	10 (14.28)	4 (5.71)	0.48
Endometrial thickness (mm) (SD)		8.15 (0.85)	8.04 (1)	
Chemical pregnancy, n (%)		24 (34.2)	12 (17.14)	0.020
Clinical pregnancy, n (%)		24 (34.2)	12 (17.14)	0.020
Occurrence of liver birth, n (%)		22 (31.4)	6 (8.6)	<0.01
Number of abortion, n (%)		2 (2.9)	6 (8.6)	0.14
Number of Gonadotropin, mean (SD)		3.5 (0.85)	3.7 (0.92)	0.18
Days of stimulation , mean (SD)		10.25 (2.8)	10.8 (4.3)	0.37

Chemical pregnancy: Positive serum Beta- subunit Human Chorionic Gonadotropin (β-hCG) in 5-6 wk after LMP last menstrual period or 13-15 days after ET.

Clinical pregnancy: Number of sac in ultrasonography for the total number of IVF cycles.

Live birth rate: The percentage of all cycles that lead to live birth (more than 20 wk)

rFSH: recombinant follicle stimulating hormone

HMG: Human menopausal gonadotropin

LH: luteinizing hormone

## Discussion

The theory that both FSH and LH are needed for the complete stimulation of follicular maturation dates back to 1959 when, Balasch proposed that action of both gonadotropins is accepted to be necessary for follicular maturation and steroid genesis (12). The supplementation of exogenous LH with FSH in controlled ovarian stimulation is essential for patients with hypogonadotropic hypogonadism (12).

Tesarik showed that supplementation with LH resulted in an increase in the number of mature oocytes and good-quality zygotes and embryos and higher implantation rates when compared with stimulation with FSH alone (13). Some investigators have reported lower estradiol biosyn-thesis, lower oocyte and embryo yield, and a higher frequency of early pregnancy loss in normogonadotrophic women down-regulated with a GnRH agonist and stimulated with highly recombinant FSH when compared with women stimulated with hMG or with a combination of hMG and FSH (14-16).

The present study compared clinical pregnancy outcomes in patients undergoing IVF/ICSI cycles using either hMG+rFSH or rFSH+rLH for COS. All patients were pituitary-suppressed using GnRH agonist protocol and fixed low dose gonadotropin. Our study is suggestive for greater number of embryo transferred and higher clinical pregnancy rate which leads to higher live birth rate in favor of hMG+rFSH regimen. We stergaard study comparing hMG vs. rFSH were indicative for a border line significant difference in favor of hMG with regard to pregnancy and live birth rates which was later confirmed by recent studies and it was claimed that hMG is superior to rFSH in terms of clinical efficiency (16). These findings has lead to the idea that the superiority of hMG originates from its LH content, thus adding recombinant LH to conventional rFSH cycles may results in the same outcomes (5).

This idea was later assessed by in vitro studies. The hMG shows two types of LH activity, one is derived from LH and the other one, which is also known to be stronger, comes from human chorionic gonadotropin (hCG) content (17). It was shown that LH and hCG bind to the same receptor, the luteinizing hormone-chorionic gonadotropin receptor because they are the same in more than 80% of amino acids sequence (18). On the other hand, LHCGR responds differently to LH and hCG which causes different effects of each molecule in human physiology during both follicle development and first trimester of pregnancy (19).

Clinical data on the LH activity of rLH in comparison to hCG contained in hMG is very scarce. In the present study, hormonal assay including serum levels of LH, FSH, AMH and prolactin was compared between study groups which showed no statistical significance. This finding is along with prior reports of Requena and colleagues indicating similarity of serum hormonal profile obtained using the combination of rFSH+rLH vs. hMG during COS (20). They concluded that steroidegenetic activity of these regimens is the same as well. Fábregues concluded that in women undergoing controlled ovarian hyperstimulation under pituitary suppression for Assisted Reproductive Technology, the recombinant combined product containing FSH and LH in a fixed 2:1 ratio is more effective than HP-hMG in terms of follicle development, oocyte yield and quality, and fertilization rates (21).

Primary studies evaluating clinical outcomes of COS with either rFSH+rLH vs. hMG were limited by their sample size and study population. Thus they reported not definite but comparable results in normo-gonadotropic women older than 35 years in terms of embryo quality, pregnancy rate, and implantation rate (5, 21). However, German IVF Registry, including more than 4000 cycles, demonstrated that pregnancy rate and implantation rate were significantly higher in rFSH+rLH preparation in comparison to both rFSH+hMG and hMG alone (3). First meta-analyses have demonstrated that hMG was not inferior to r-FSH with regard to pregnancy and live birth rates (16).

The Coomarasamy A Cochrane review confirmed these data, finding a border-line significant difference of a 5% higher clinical pregnancy rate in women stimulated with menotrophins (27%) compared with FSH (22%). Recent meta-analyses and reviews demonstrated that hMG is superior to rFSH with regard to clinical efficiency. Coomarasamy concluded his review claiming that the clinical superiority of hMG is because of the LH it contains, than it might be possible to add recombinant LH to achieve the same results (22).

Our finding confirm the hypothesis that treatment with hMG plus rFSH could achieve the same results in the number of oocyte, Number of M2 oocyte and embryo quality, but we find a statistical difference in chemical and clinical pregnancy and live birth rate with a better embryo quality in the second group (hMG+rFSH). This difference has leveled because of the total number and quality of embryo which is higher in hMG+rFSH group, although the quality of embryo difference in the two groups was not significant. Also the number of study subtypes was limited (23).

An interesting finding was reported by Revelli *et al* (9). A total number of 848 IVF patients with the same base line characteristics were recruited in a real life population study. In their study, authors were able to compare subgroups having the same oocyte yield but treated with either rFSH+rLH or hMG. In our study we did not registered the costs of each IVF cycle using rFSH+rLH or hMG+rFSH. But given the lower number of medication administered in hMG preparation it seems that this regimen would cost much less than rFSH+rLH. Future studies are warranted to exactly compare the costs of each regimen.

## Conclusion

In conclusion our results are suggestive for better clinical pregnancy rate and live birth rate using hMG+rFSH in pituitary-suppressed patients undergoing IVF/ICSI. But it is necessary to implement studies on more patients in randomized clinical trials so these results are confirmed. Also future studies must be done in terms of response to treatment with any of the methods hMG+r-FSH or rFSH+rLH in chronic medical conditions such as polycystic-ovarian syndrome or endometriosis. 

## Note

***Registration ID in IRCT: IRCT2016092720351N3***

***This article extracted from Residential thesis. (Mina Farshbaf taghinejad)***
